# Safety findings from the phase 1/2 MOSAIC study of miransertib for patients with PIK3CA-related overgrowth spectrum or Proteus syndrome

**DOI:** 10.1186/s13023-025-03831-z

**Published:** 2025-07-25

**Authors:** Whitney Eng, Ionela Iacobas, Jonathan Perkins, Giuseppe Zampino, Chiara Leoni, Paola Sabrina Buonuomo, Alessandra Simonetti, Himanshu Goel, Michael Briones, Mo Huang, Gregory Goldmacher, Danny Liaw, Adrienne Hammill

**Affiliations:** 1 Boston Children’s Hospital and the Dana-Farber Cancer Institute, Harvard Medical School, Boston, MA USA; 2 Seattle Children’s Hospital, 4800 Sandpoint Way NE, Seattle, WA 98115 USA; 3 Texas Children’s Hospital/Baylor College of Medicine, Houston, TX USA; 4 Università Cattolica del Sacro Cuore, Rome, Italy; 5 Center for Rare Diseases and Birth Defects, Department of Woman and Child Health and Public Health, Fondazione Policlinico Universitario A. Gemelli, Rome, Italy; 6 Ospedale Pediatrico Bambino Gesù, Rome, Italy; 7 School of Pediatrics, University of Rome “Tor Vergata”, Rome, Italy; 8 Hunter Genetics, Waratah, NSW 2298 Australia; 9 University of Newcastle, Callaghan, NSW 2308 Australia; 10 School of Medicine and Aflac Cancer and Blood Disorder Center, Emory University, Atlanta, GA USA; 11 Merck & Co., Inc., Rahway, NJ USA; 12 Cincinnati Children’s Hospital Medical Center and the University of Cincinnati College of Medicine, Cincinnati, OH USA

**Keywords:** Miransertib, *AKT*, *PIK3CA*, Proteus syndrome, *PIK3CA*-related overgrowth spectrum

## Abstract

**Background:**

*PIK3CA*-related overgrowth spectrum (PROS) and Proteus syndrome are associated with mosaic tissue overgrowth of varying severity that commonly presents in childhood. The multicenter, open-label, phase 1/2 MOSAIC study (NCT03094832) was designed to evaluate the clinical efficacy and safety of the selective pan-AKT inhibitor miransertib for participants with PROS or Proteus syndrome.

**Methods:**

Participants ≥ 2 years of age with PROS with documented somatic *PIK3CA* mutations or Proteus syndrome with documented somatic *AKT1* mutations were enrolled to receive oral miransertib at a starting dose of 15 mg/m^2^ every day for the first 3 cycles (1 cycle = 28 days) and miransertib 25 mg/m^2^ every day thereafter, provided no clinically significant drug-related toxicities were observed. The initial primary objective of the study was to assess clinical response to miransertib. Due to study design and data collection limitations, evaluating efficacy was no longer considered feasible and the primary objective was updated in 2021 to evaluate the safety and tolerability of miransertib.

**Results:**

Between May 16, 2017 and January 25, 2021, 49 participants were enrolled and received ≥ 1 dose of study drug, comprising the safety analysis population. Forty-five participants had a diagnosis of PROS and four had a diagnosis of Proteus syndrome. The median (range) age at enrollment was 7 years (2–41). Median (range) duration of treatment was 20.5 months (9.9–45.6). A total of 23 (46.9%) participants had a drug-related adverse event, most commonly decreased neutrophil count (n = 6, 12.2%), increased blood insulin (n = 5, 10.2%), and stomatitis (n = 5, 10.2%). One (2.0%) participant experienced a grade 3 drug-related adverse event (deep vein thrombosis). No drug-related adverse events led to early study discontinuation or death. Laboratory assessment values remained generally stable throughout the study.

**Conclusion:**

Miransertib was safe and tolerable in participants with a confirmed diagnosis of PROS or Proteus syndrome. Future investigations are needed to determine whether patients receive measurable clinical benefit from miransertib.

*Trial Registration*: NCT03094832 registered Mar 28, 2017, https://clinicaltrials.gov/ct2/show/NCT03094832.

**Supplementary Information:**

The online version contains supplementary material available at 10.1186/s13023-025-03831-z.

## Background

*PIK3CA*-related overgrowth spectrum (PROS) and Proteus syndrome are rare somatic overgrowth disorders caused by a single gain-of-function mutation in the *PIK3CA* or *AKT1* genes, respectively [1–3]. PROS and Proteus syndrome are characterized by asymmetric organ and tissue overgrowth of varying severity depending on the extent of the mosaicism. Both disorders are associated with severe vascular malformations and debilitating musculoskeletal issues, and often confer a poor prognosis [1, 4]. Historically, treatment for both disorders relied solely on surgical or interventional procedures or best supportive care.

Recently, targeted medical therapies have been introduced for the treatment of a variety of overgrowth disorders caused by mutations in the PI3K-AKT-mTOR pathway. Sirolimus, an oral mTOR inhibitor, was found to be safe and effective in a single-arm phase 2 study of patients with complex vascular anomalies [5]. Sirolimus has been investigated as upfront treatment for a variety of PROS disorders, often treating abnormal growth as well as the symptoms of pain, infection, and bleeding [3, 5, 6]. In a pooled study of 30 patients with PROS who received low-dose sirolimus across three centers, treatment resulted in reduction in tissue volume in disease-affected areas but not in healthy tissues [6], although several clinically important adverse events were observed.

Alpelisib, an oral PIK3CA inhibitor first approved for use in breast cancer, was repurposed for use in patients with vascular anomalies via a compassionate use program. A retrospective chart review study (EPIK-P1) evaluated 57 patients who received alpelisib under this program. Patients were 2 years old or greater and had a diagnosis of PROS, confirmed *PIK3CA* mutation, and severe or life-threatening conditions [7]. Patients experienced improvement in PROS-related symptoms (including fatigue, vascular malformation, limb asymmetry, and disseminated intravascular coagulation). Among 32 patients who had available imaging, 37.5% had at least 20% reduction in the volume of target lesions. The drug was tolerated well, with 38.6% of patients experiencing a drug-related adverse event; no drug-related deaths occurred. Based on this study, alpelisib gained accelerated approval in the United States for the treatment of adult and pediatric patients at least 2 years of age with severe manifestations of PROS who require systemic therapy.

Miransertib is an orally administered, potent, and selective allosteric pan-AKT inhibitor shown to prevent and reduce PI3K mutation-associated vascular malformations in mouse models [8]. Several case reports have described symptomatic improvement and reduction of overgrowth with miransertib in patients with Proteus syndrome or PROS [9–11].

The multicenter, open-label, phase 1/2 MOSAIC study was designed to evaluate the clinical efficacy and safety of miransertib (also known as ARQ 092 or MK-7075) for participants with PROS or Proteus syndrome. Limitations in the original study design and methodology precluded meaningful analysis of changes in disease burden, and the primary objective of the study was amended to safety and tolerability only in 2021. Thus, we present the safety analysis from the MOSAIC study of miransertib for participants with PROS or Proteus syndrome.

## Methods

### Study design/participants

The open-label, phase 1/2 MOSAIC study (MK-7075-002; NCT03094832) was conducted at 11 sites in Australia, Italy, Spain, and the United States. According to the initial protocol, eligible participants were at least 2 years old, had body surface area (BSA) of ≥ 0.33 m^2^, had a clinical diagnosis of PROS or Proteus syndrome with documented *PIK3CA* or *AKT1* somatic mutations, respectively, and measurable disease (defined as at least 1 lesion that could be accurately measured by magnetic resonance imaging [MRI] for PROS or standardized digital photography for Proteus syndrome). Participants had poor prognosis, significant morbidity, and/or clinically progressive or worsening disease (defined as an increase in the number or size of overgrowth lesions in the past 12 months by investigator assessment). Other key inclusion criteria were adequate organ function as defined by the protocol and availability of a fresh or archival overgrowth lesion tissue sample. Participants were excluded if they had previous intolerance to or severe toxicity attributed to AKT inhibitors, any experimental systemic therapy for PROS or Proteus syndrome within 2 weeks of the first miransertib dose, or major surgical procedures or other locoregional therapy (e.g., radiotherapy) within 4 weeks of the first miransertib dose.

Inclusion criteria were updated in a protocol amendment (March 7, 2019) to set an upper age limit of 30 years, with disease progression or worsening within the past 6 months (Cohorts 1–3). Cohort 1 included participants with PROS with documented somatic *PIK3CA* mutations; Cohort 2 included participants with Proteus syndrome with documented somatic *AKT1* mutations; Cohort 3 included participants with PROS or Proteus syndrome who did not meet the eligibility criteria for either Cohorts 1 or 2. Additional participants with PROS or Proteus syndrome who were previously or currently receiving miransertib under the Compassionate Use/Expanded Access Program were eligible for enrollment into Cohort 4.

All participants provided written informed consent and, where applicable, signed assent. The protocol and all amendments were approved by the appropriate institutional review board or independent ethics committee at each site. The study was conducted according to Good Clinical Practice guidelines and the principles of the Declaration of Helsinki. An external Data Monitoring Committee monitored participants’ safety by reviewing and evaluating study data, conduct, and progress at least twice per year.

### Treatment

Participants received miransertib 15 mg/m^2^ orally daily for 3 cycles (with each cycle lasting 28 days) and were dose-escalated to a dose of 25 mg/m^2^ orally daily, provided no clinically significant drug-related toxicity had been observed. Notably, the original trial protocol permitted a second dose escalation to 35 mg/m^2^ orally daily; after the first six participants were enrolled, the protocol was amended to permit a single escalation to 25 mg/m^2^ orally daily due to concerns about the tolerability of the 35 mg/m^2^ dose. Participants in Cohort 4 who were already receiving miransertib at the time of enrollment were continued on the same dose without exceeding 25 mg/m^2^ daily. Dose was re-evaluated at each study visit based on BSA and adjusted as necessary. Dose delays and reductions were permitted to manage miransertib-related toxicity; once reduced, re-escalation of the dose was not permitted. Treatment continued for up to 48 cycles or until one of the following occurred: disease progression, unacceptable toxicity, the start of a new investigational agent, the development of a concurrent medical condition that precluded or contraindicated further study drug administration, serious protocol violation, or the decision to discontinue treatment by the investigator, participant, parent, or guardian.

### Outcomes, assessments, and analysis

According to the original MOSAIC study design, the primary objective was to obtain evidence of the clinical activity of miransertib in participants with PROS or Proteus syndrome, as assessed by change in lesion size or volume from baseline and/or change in body measurements from baseline. Imaging was to occur at Screening, cycles 7, 13, 19, 25, 37, and end of treatment. Imaging assessments of lesions were performed using MRI, although other imaging modalities such as computed tomography scans, ultrasound, dual-energy X-ray absorptiometry, and photography were permitted. Image acquisition protocols were not standardized across sites and timepoints, contributing to the inability to compare efficacy for a given participant or across participants. At the time of study design, defined assessment rules for response were not available for PROS or Proteus syndrome due to the heterogeneity of the diseases and the uniqueness of each patient. Response evaluation was protocol-defined as ≥ 20% decrease in target lesion from baseline (for PROS) and ≤ 5% increase from baseline in total CCTN (in Proteus syndrome). Nevertheless, the lack of easily defined borders between healthy and disease-affected tissue hampered reliable and reproducible response evaluation, making it impossible to meaningfully assess changes in disease burden as a measure of miransertib efficacy.

As a result, the primary endpoint of the study was amended to safety and tolerability of miransertib in participants with PROS and Proteus syndrome on March 5, 2021. Safety and tolerability were assessed based on the frequency and severity of adverse events from the first dose until 90 days after the last dose of study drug. Adverse events were graded according to the Common Terminology Criteria for Adverse Events (CTCAE) Version 4.03. Attribution of adverse events related to study drug was determined by the investigator and adverse events were reported as related to miransertib regardless of whether they were considered definitely, probably, or possibly related to study drug. Clinical laboratory tests included hematology, blood chemistry, and urinalysis. Samples for fasting clinical blood tests were collected at screening and at pre-specified scheduled visits (cycles 2–5, every odd cycle up until the end of year 1, every third cycle in years 2–4 starting at cycle 16, at end of treatment, and at the 30-day safety follow-up visit) at the local laboratory of the investigational site. Samples for insulin and glucose blood tests were collected at screening and were pre-specified for collection at cycles 7, 13, 19, 25, 37 and at the end of treatment.

Safety and tolerability were evaluated in the safety population, comprised of all participants who received ≥ 1 dose of study drug.

## Results

### Participant baseline characteristics

**Fig. 1:**
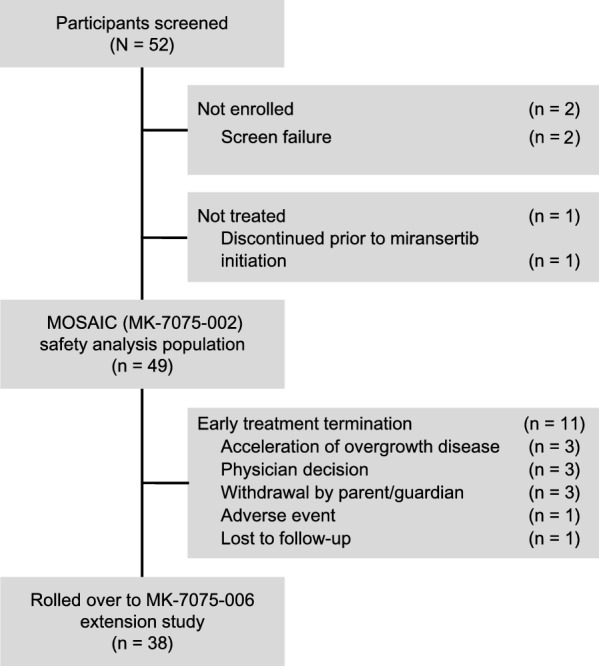
Study profile

Between May 16, 2017 and January 25, 2021, 52 participants were screened and 50 were enrolled in the MOSAIC study. A total of 49 participants received ≥ 1 dose of study drug and were included in the safety analysis population, including 17 participants prior to the March 7, 2019 amendment, 22 participants in Cohort 1, 1 in Cohort 2, 8 in Cohort 3 and 1 in Cohort 4. A total of 11 (22.4%) participants stopped study drug early (Fig. 1). Beginning on November 2, 2021, the remaining 38 participants were rolled over into the MK-7075–006 extension study for safety follow-up upon the termination of the MOSAIC study by the sponsor. As of May 26, 2022, the median time from initiation of study therapy to the data cutoff date was 27.4 months (range, 15.5–59.9). The median age of the study population at enrollment was 7 years (range, 2–41), with 7 (14.3%) participants who were ≥ 18 years of age (Table 1). Approximately half of the participants (n = 27, 55.1%) were male. Most participants (n = 45, 91.8%) had a diagnosis of PROS, including 24 (49.0%) participants with CLOVES syndrome, 10 (20.4%) participants with lymphatic malformation, and 7 (14.3%) participants with Klippel-Trenaunay syndrome. Four additional participants had a diagnosis of Proteus syndrome. The median time from diagnosis to study drug initiation for the entire cohort was 5.2 years. Median duration of treatment was 20.5 months (range, 9.9–45.6) for 48 of the 49 participants (1 participant was lost to follow-up after approximately 7 cycles of study drug and did not have an available date of last drug administration) (Table S1). A total of 48 participants received miransertib at a dose of 15 mg/m^2^, of whom 47 were escalated to a dose of 25 mg/m^2^ per the study treatment plan. One participant had a dose escalation to 35 mg/m^2^ (per the maximum dose allowed by the original study protocol) for the treatment of overgrowth, lipomatosis, and macroglossia. One participant had a dose reduction to 10 mg/m^2^ due to grade 2 dizziness, and a subsequent dose escalation to 15 mg/m^2^ after the dizziness resolved. One participant who was initially treated with miransertib through the Compassionate Use Program entered Cohort 4 on their prior dose of 25 mg/m^2^.

**Table 1:** Demographic and baseline disease characteristics in the safety population

Participant characteristics	Overall, N = 49
*Age at study inclusion, years*	
Median (range)	7.0 (2 to 41)
Mean (SD)	10.0 (8.30)
≥ 18 years	7 (14.3)
*Sex*	
Male	27 (55.1)
Female	22 (44.9)
*Race*	
Asian	4 (8.2)
Black or African American	6 (12.2)
Multiple	1 (2.0)
White	38 (77.6)
*BSA, m*^*2*^	
Median (range)	0.97 (0.49 to 2.23)
Mean (SD)	1.14 (0.52)
*Disease diagnosis*	
PROS*	45 (91.8)
CLOVES syndrome	24 (49.0)
Lymphatic malformation	10 (20.4)
Klippel-Trenaunay syndrome	7 (14.3)
Fibroadipose hyperplasia	1 (2.0)
Hemimegalencephaly	1 (2.0)
Type I macrodactyly and muscle hemihypertrophy	1 (2.0)
Unspecified overgrowth†	1 (2.0)
Proteus syndrome	4 (8.2)
*Life stage at which overgrowth was reported*	
Prenatal	11 (22.4)
At birth	18 (36.7)
Infancy	12 (24.5)
Childhood	8 (16.3)
*Time since diagnosis (years)*	
Median (range)	5.20 (− 0.1 to 41.5)
Mean (SD)	8.05 (8.296)

Data are n (%) except where otherwise specified. BSA, body surface area; CLOVES, congenital lipomatous overgrowth, vascular malformations, epidermal nevi, spinal/skeletal anomalies/scoliosis; SD, standard deviation

^*^Participants were counted once based on primary subtype entered into the study database. Participants had to have at least 2 spectrum or 3 isolated features of PROS in addition to presence of *PIK3CA* mutation to be included in the study; therefore, participants may have presented with additional subtypes

^†^Met criteria for sporadic and mosaic overgrowth, with patchy and irregular vascular malformations

A total of 23 (46.9%) participants out of 49 had a drug-related adverse event (Table 2), most commonly decreased neutrophil count (n = 6, 12.2%), increased blood insulin (n = 5, 10.2%), and stomatitis (n = 5, 10.2%) (Table 3). One (2.0%) participant experienced a grade 3 drug-related adverse event (deep vein thrombosis) after over one year of study therapy, with noted potential confounding factors of “COVID-19 immunization” and “chest infection”. No serious adverse events and no adverse events leading to early study discontinuation or death were attributed to study drug by the investigators.

**Table 2:** Summary of adverse events by dose* in the safety population

	Overall N = 49	10 mg/m^2^ n = 1	15 mg/m^2^ n = 48	25 mg/m^2^ n = 48	35 mg/m^2^ n = 1
Any event, n (%)	44 (89.8)	1 (100.0)	38 (79.2)	41 (85.4)	1 (100.0)
Any grade ≥ 3 event	12 (24.5)	0	3 (6.3)	12 (25.0)	1 (100.0)
Any serious event	8 (16.3)	0	3 (6.3)	4 (8.3)	1 (100.0)
Any event leading to early study withdrawal	2 (4.1)	0	0	1 (2.1)	1 (100.0)
Any event leading to death	1 (2.0)	0	0	0	1 (100.0)
Any treatment-related event†, n (%)	23 (46.9)	0	12 (25.0)	20 (41.7)	0
Any grade ≥ 3 treatment-related event	1 (2.0)	0	0	1 (2.1)	0
Any serious treatment-related event	0	0	0	0	0
Any treatment-related event leading to early study withdrawal	0	0	0	0	0
Any treatment-related event leading to death	0	0	0	0	0

Data are n (%)

^*^Participants who received multiple doses over the course of the study are counted in the population count for each dose received, and their events are counted according to the dose at adverse event onset. As such, participants may appear in more than one dose column

†A treatment-related adverse event was defined as any adverse event considered related, probably related, or possibly related to miransertib treatment per investigator

**Table 3:** Treatment-related adverse events occurring in ≥ 5% of participants in the safety population

	Overall N = 49
Any event, n (%)	23 (46.9)
Decreased neutrophil count	6 (12.2)
Blood insulin increased	5 (10.2)
Stomatitis	5 (10.2)
Decreased appetite	4 (8.2)
Lymphocyte count decreased	4 (8.2)
Nausea	4 (8.2)
Diarrhea	3 (6.1)
Dizziness	3 (6.1)
Fatigue	3 (6.1)

Data are n (%)

Among the 49 participants in the safety population, 44 (89.8%) experienced any adverse event and 12 (24.5%) experienced a grade ≥ 3 adverse event (Table 2), regardless of relation to study medication. The most common adverse events of any cause and any grade were pyrexia (n = 20, 40.8%), vomiting (n = 19, 38.8%), and cough (n = 16, 32.7%) (Table 4). A total of 8 (16.3%) participants experienced a serious adverse event, most commonly cellulitis (n = 3, 6.1%) and anemia (n = 2, 4.1%). One (2.0%) case each of dehydration, diarrhea, febrile convulsion, mouth hemorrhage, nausea and vomiting met serious event criteria (Table S2). Three (6.3%) and 4 (8.3%) of 48 participants developed a serious adverse event while receiving miransertib at doses of 15 mg/m^2^ and 25 mg/m^2^, respectively. One participant died while on the study. The participant had history of lipomatous overgrowth, macroglossia, epilepsy, and severe neurocognitive delay. Prior surgeries included tracheostomy due to airway compression and partial glossectomy to reduce bulk of the facial mass. They had been receiving miransertib at 35 mg/m^2^ dose per the study protocol-planned escalation prior to the amendment and were tolerating the treatment well. The patient had been treated for 21 months prior to development of respiratory infection and fever, for which they received antibiotic therapy and miransertib was stopped. The participant also experienced diarrhea and vomiting and died from suspected dehydration 2 days after the last miransertib dose. This was reported as a fatal serious adverse event not considered related to study drug by the investigator. Further details are not available as the participant died at home. Additional episodes of fever, vomiting and diarrhea had occurred in the same participant and resolved with supportive care within a month prior to the fatal event.

**Table 4:** Adverse events occurring in ≥ 10% of participants in the safety population regardless of attribution to study drug

	Overall N = 49
Any event, n (%)	44 (89.8)
Pyrexia	20 (40.8)
Vomiting	19 (38.8)
Cough	16 (32.7)
Diarrhea	14 (28.6)
Abdominal pain	13 (26.5)
Upper respiratory tract infection	11 (22.4)
Constipation	10 (20.4)
Pain in extremity	10 (20.4)
Nausea	8 (16.3)
Decreased neutrophil count	8 (16.3)
Oropharyngeal pain	8 (16.3)
Arthralgia	7 (14.3)
Dizziness	7 (14.3)
Fatigue	7 (14.3)
Headache	7 (14.3)
Lymphocyte count decreased	7 (14.3)
Stomatitis	7 (14.3)
Blood lactate dehydrogenase increased	6 (12.2)
Decreased appetite	6 (12.2)
Rash maculopapular	6 (12.2)
Anemia	5 (10.2)
Blood insulin increased	5 (10.2)
Cellulitis	5 (10.2)
Dry skin	5 (10.2)
Epistaxis	5 (10.2)
Hyperkalemia	5 (10.2)
Nasal congestion	5 (10.2)
Pharyngitis	5 (10.2)
Platelet count decreased	5 (10.2)
Rhinitis	5 (10.2)
White blood cell count decreased	5 (10.2)

Data are n (%)

Laboratory test results that met the criteria for a grade ≥ 1 adverse event are summarized for all participants who had ≥ 1 post-baseline laboratory assessment. Transient elevations in aspartate aminotransferase (AST) were the most common event (grade 1, n = 25/49, 51.0%; grade 2, n = 1/49, 2.0%) (Table 5). Transient increased levels of other liver enzymes were also observed, including bilirubin (grade 1, n = 10/48, 20.8%; grade 2, n = 4/48, 8.3%), alanine aminotransferase (ALT) (grade 1, n = 9/49, 18.4%; grade 2, n = 2/49, 4.1%), and alkaline phosphatase (grade 1, n = 2/47, 4.3%; grade 2, n = 1/47, 2.1%). Key laboratory assessment values at prespecified time points, including blood glucose and hemoglobin A1C, are shown in Figure S1.

**Table 5:** Post-baseline laboratory values that met CTCAE criteria in the safety population

Lab value*	Overall N = 49
Alkaline phosphatase (n = 47)	
Grade 1	2 (4.3)
Grade 2	1 (2.1)
Grade 3	0
Grade 4	0
Alanine aminotransferase (n = 49)	
Grade 1	9 (18.4)
Grade 2	2 (4.1)
Grade 3	0
Grade 4	0
Aspartate aminotransferase (n = 49)	
Grade 1	25 (51.0)
Grade 2	1 (2.0)
Grade 3	0
Grade 4	0
Bilirubin (n = 48)	
Grade 1	10 (20.8)
Grade 2	4 (8.3)
Grade 3	0
Grade 4	0

Data are n (%)

*n represents the number of participants with ≥1 post-baseline measurement

## Discussion

The phase 1/2 MOSAIC study investigated the selective allosteric pan-AKT inhibitor miransertib as a potential treatment for participants with confirmed PROS or Proteus syndrome. The effects of miransertib on disease burden could not be meaningfully analyzed due to lack of standardization of image acquisition methods across participants and timepoints. The protocol was amended and participants were rolled over into a long-term safety extension study. Miransertib was generally well tolerated across the 49 participants who received study drug and comprised the safety analysis population. The majority of drug-related adverse events were of low-grade and were expected based on prior observations of AKT inhibitors. Laboratory assessments indicated generally stable levels of liver enzymes, blood glucose, and hemoglobin A1C over time. The safety profile of miransertib in participants with PROS or Proteus syndrome in the MOSAIC study corresponded with prior observations of the drug for other applications [9, 10, 12]. Adverse events related or possibly related to miransertib reported in the literature include elevated liver enzymes, elevated blood glucose, and dry mouth. Miransertib treatment was also reported to be feasible and tolerable, and continued for over a year in all described patients [9–12]. Although no direct comparison between study results can be made, the safety profile of miransertib may be more favorable than that of sirolimus. In a study of 39 participants with PROS treated with sirolimus, 37% of drug-related adverse events were of grade 3–4 severity and 18% of participants had discontinued sirolimus treatment due to the associated toxicity [6]. In contrast, in the MOSAIC study, miransertib-related adverse events included only 1 occurrence (2.0%) of grade 3 severity, and no events that led to participant discontinuation of drug treatment.

Although the MOSAIC study was originally initiated as a dose-finding, safety, and tolerability study that also included efficacy endpoints, the efficacy endpoints were removed due to operational and design barriers preventing their accurate assessment. Of note, 38 out of 49 participants in the study chose to continue on miransertib therapy as part of the MK-7075–006 extension study for safety follow-up at the end of the MOSAIC study, even after additional agents such as alpelisib became available. This observation suggests these participants viewed the efficacy and safety outcomes of miransertib therapy as favorable. Based on this experience, we recommend that future studies implement a standard protocol for MRI of affected anatomic regions to enable reliable evaluation of efficacy. Due to overlapping clinical features among patients with the genetic mutations defining PROS or Proteus syndrome, central review of patient diagnosis may also be an important consideration for future studies. Unique difficulties exist in measuring heterogenous overgrowth in patients with Proteus syndrome and PROS, highlighting a role for central imaging collection and assessment with real-time enforcement of image acquisition guidelines. Finally, evaluation of additional endpoints may be prudent for future trials, as patients with overgrowth syndromes often exhibit symptoms that are not captured by radiologic assessment alone.

Case studies have demonstrated that patients with Proteus syndrome or PROS experience clinical benefit from miransertib treatment, including reduction in the size of overgrowth lesions. Patients also report improved quality of life, improved mobility, and reduction in seizure frequency [9–12]. The National Institute of Health initiated a multicohort, single-arm, dose-escalation, phase 2 study of miransertib for participants with Proteus syndrome in 2020 that is currently ongoing. The primary endpoint in this study is the proportion of participants with response (defined as having a CCTN growth rate [expressed as surface area/26 cycles] below a prespecified rate), and secondary endpoints include quality of life, long-term safety and tolerability, and duration of response. The study plans to enroll approximately 45 participants and may provide more definitive evidence for the utility of miransertib in the treatment of somatic overgrowth disorders.

## Conclusion

The results of this study demonstrate that miransertib is safe and tolerable in patients with PROS or Proteus syndrome. These results warrant further investigation of efficacy, optimal dosing, and long-term safety. This is particularly important given that many patients initiate these medications at a young age and may need to take them chronically to prevent recurrence of complications. Patients with overgrowth disorders experience unique morbidity, and this study marks an important development in treating mosaic overgrowth syndromes by targeting the specific pathways that drive disease progression. Future trials of targeted therapies may focus on distinct response criteria and addressing this unmet need.

## Supplementary Information

Supplementary material 1.

## Data Availability

Merck Sharp & Dohme LLC, a subsidiary of Merck & Co., Inc., Rahway, NJ, USA (MSD) is committed to providing qualified scientific researchers access to anonymized data and clinical study reports from the company’s clinical trials for the purpose of conducting legitimate scientific research. MSD is also obligated to protect the rights and privacy of trial participants and, as such, has a procedure in place for evaluating and fulfilling requests for sharing company clinical trial data with qualified external scientific researchers. The MSD data sharing website (available at: https://externaldatasharing-msd.com/) outlines the process and requirements for submitting a data request. Applications will be promptly assessed for completeness and policy compliance. Feasible requests will be reviewed by a committee of MSD subject matter experts to assess the scientific validity of the request and the qualifications of the requestors. In line with data privacy legislation, submitters of approved requests must enter into a standard data-sharing agreement with MSD before data access is granted. Data will be made available for request after product approval in the US and EU or after product development is discontinued. There are circumstances that may prevent MSD from sharing requested data, including country or region-specific regulations. If the request is declined, it will be communicated to the investigator. Access to genetic or exploratory biomarker data requires a detailed, hypothesis-driven statistical analysis plan that is collaboratively developed by the requestor and MSD subject matter experts; after approval of the statistical analysis plan and execution of a data-sharing agreement, MSD will either perform the proposed analyses and share the results with the requestor or will construct biomarker covariates and add them to a file with clinical data that is uploaded to an analysis portal so that the requestor can perform the proposed analyses.

## Abbreviations

AKT: Protein kinase B
BSA: Body surface area
CCTN: Cerebriform connective tissue nevus
CLOVES: Congenital lipomatous overgrowth, vascular malformations, epidermal nevis, spinal/skeletal anomalies/scoliosis
CTCAE: Common terminology criteria for adverse events
MRI: Magnetic resonance imaging
mTOR: Mammalian target of rapamycin
PI3K: Phosphoinositide-3-kinase
PROS: *PIK3CA*-Related overgrowth spectrum
PTEN: Phosphatase and tensin homolog
SD: Standard deviation
