# Supplementary material for: Safety findings from the phase 1/2 MOSAIC study of miransertib for patients with PIK3CA-related overgrowth spectrum or Proteus syndrome
Source: Orphanet J Rare Dis. 2025 Jul 25;20:375. doi: 10.1186/s13023-025-03831-z (PMC12296598; doi:10.1186/s13023-025-03831-z)
Supplement: Supplementary file 1 — Supplementary material 1. [file 13023_2025_3831_MOESM1_ESM.docx]

Supplementary Appendix for **Safety findings from the phase 1/2 MOSAIC study of miransertib for patients with *PIK3CA*-related overgrowth spectrum or Proteus syndrome**. Authors: Eng et al.

**Table of Contents**

| **Title** | **Page No.** |
| --- | --- |
| Figure S1. Changes in key lab values from baseline | 2-4 |
| Table S1. Summary of treatment exposure | 5 |
| Table S2. Serious adverse events of any cause | 6 |

**Figure S1. Changes in key lab values over time in the safety population.**

Mean (SD) alanine aminotransferase levels (A), aspartate aminotransferase (B), alkaline phosphatase (C), bilirubin (D), blood glucose (E), hemoglobin A1C (F) at baseline and prespecified time points for Cohorts 1-3. C=Cycle, D=Day, EOT=End of treatment

**A.**

**B.**

**C.**

**D.**

**E.**

**F.^a^**

^a^One additional participant had a recorded result of hemoglobin A1C of 92.0% at end of treatment, likely a data entry error, and was not included in the graph.

**Table S1. Summary of treatment exposure in the safety population.**

| **Participant characteristics** | **Overall N = 49*** |
| --- | --- |
| Duration of treatment, months |  |
| Median (range) | 20.5 (9.9−45.6) |
| Mean (SD) | 23.5 (10.7) |
| Relative daily miransertib dose received, n (%)† |  |
| 10 mg/m^2^ | 1 (2.0) |
| 15 mg/m^2^ | 48 (98.0) |
| 25 mg/m^2^ | 48 (98.0) |
| 35 mg/m^2^ | 1 (2.0) |

*One participant was missing the date of last exposure and measures of treatment exposure could not be calculated for them. Therefore, duration of treatment was calculated based on 48 participants.

†Participants who received multiple dose intensities of miransertib are counted once for each dose received.

SD, standard deviation.

**Table S2. Serious adverse events in the safety population regardless of attribution to study drug.**

|  | **Overall N = 49** | **Related to drug** |
| --- | --- | --- |
| **Any event, n (%)** | 8 (16.3) | 0 |
| Cellulitis | 3 (6.1) | 0 |
| Anemia | 2 (4.1) | 0 |
| Dehydration | 1 (2.0) | 0 |
| Diarrhea | 1 (2.0) | 0 |
| Febrile convulsion | 1 (2.0) | 0 |
| Mouth hemorrhage | 1 (2.0) | 0 |
| Nausea | 1 (2.0) | 0 |
| Vomiting | 1 (2.0) | 0 |

Data are n (%).
